# The Predictive Role of Thiol/Disulfide Homeostasis as an Oxidative Stress Parameter in Sarcopenic Obesity

**DOI:** 10.3390/medicina61091708

**Published:** 2025-09-19

**Authors:** Ayse Dikmeer, Funda Eren, Salim Neselioglu, Zeynep Sahiner, Merve Hafizoglu, Didem Karaduman, Cansu Atbas, Ibrahim Ileri, Burcu Balam Dogu, Mustafa Cankurtaran, Filiz Akbiyik, Ozcan Erel, Meltem Gulhan Halil

**Affiliations:** 1 Division of Geriatrics, Department of Internal Medicine, Faculty of Medicine, Hacettepe University, 06230 Ankara, Türkiye; 2 Department of Clinical Biochemistry, Ankara Bilkent City Hospital, 06800 Ankara, Türkiye; 3 Department of Clinical Biochemistry, Faculty of Medicine, Yıldırım Beyazit University, 06800 Ankara, Türkiye; 4 Enterprise Services & Solutions, Siemens Healthineers Laboratory, Ankara Bilkent City Hospital, 06800 Ankara, Türkiye

**Keywords:** older adults, oxidative stress, sarcopenic obesity, thiol/disulfide homeostasis

## Abstract

*Background and Objectives:* Sarcopenic obesity (SO), characterized by the coexistence of excess adiposity and reduced muscle mass/function, is associated with adverse outcomes in older adults. Oxidative stress has been implicated in the pathogenesis of both obesity and sarcopenia. This study aimed to evaluate the association between thiol/disulfide homeostasis (TDH), ischemia-modified albumin (IMA), and SO in obese older adults. *Materials and Methods:* In this cross-sectional study, 132 obese individuals aged ≥65 years were enrolled from a geriatrics outpatient clinic. SO was defined based on the ESPEN/EASO criteria, incorporating anthropometric, body composition, and muscle function measures. Serum native and total thiol levels, disulfide concentrations, and IMA were assessed. Logistic regression identified independent predictors of SO, and ROC analysis evaluated the discriminatory power of oxidative parameters. *Results:* SO was present in 15.2% (*n* = 20) of participants. Patients with SO exhibited significantly lower native (*p* = 0.003) and total thiol levels (*p* < 0.001), and higher disulfide/native thiol (*p* = 0.009) and disulfide/total thiol ratios (*p* = 0.009). IMA levels were slightly elevated in SO but not significantly different (*p* = 0.13). In multivariable regression, age and disulfide/native thiol ratio were independent predictors of SO (OR = 5.71, *p* = 0.041). ROC analysis showed that disulfide/native thiol ratio had moderate predictive accuracy (AUC = 0.684, *p* = 0.008), with a cut-off > 6.63 yielding 92.86% specificity. *Conclusions:* Older adults with SO exhibit disrupted redox balance, as evidenced by altered TDH parameters. The disulfide/native thiol ratio may serve as a useful oxidative biomarker for identifying SO. These findings highlight the potential role of oxidative stress in SO and warrant further research into targeted antioxidant strategies.

## 1. Introduction

Sarcopenic obesity (SO), defined as the coexistence of low skeletal muscle mass/function and excess adiposity, represents a significant geriatric syndrome with profound implications for morbidity, disability, and mortality in older adults [1,2]. The dual burden of adiposity and muscle wasting results in worse clinical outcomes than sarcopenia or obesity alone, including increased frailty, cardiovascular disease, and all-cause mortality [3,4]. Skeletal muscle mass exhibits a progressive decline with advancing age, with well-established differences across sex and age groups; this age-related muscle loss plays a pivotal role in the development of sarcopenic obesity [5].

The pathophysiology of SO is multifactorial and includes anabolic resistance, chronic inflammation, hormonal alterations, insulin resistance, and mitochondrial dysfunction [6,7]. Among these, oxidative stress has emerged as a critical contributor to SO, as it disrupts redox-sensitive signaling pathways and exacerbates both adipose- and muscle-related metabolic derangements [8,9].

Thiol/disulfide homeostasis (TDH) is a recently established and dynamic marker of oxidative stress, reflecting the equilibrium between reduced thiols (-SH) and oxidized disulfide bonds (-S-S-) in plasma [10]. In healthy individuals, TDH is tightly regulated, but disruptions in this balance are indicative of oxidative damage and have been implicated in a variety of age- and metabolism-related diseases including diabetes, inflammatory disorders, and sarcopenia [11,12].

In sarcopenic and osteosarcopenic populations, studies have demonstrated significantly lower native and total thiol levels and elevated disulfide ratios, indicating an oxidative shift in TDH [12,13]. Obesity has also been associated with disturbed TDH, even in the absence of insulin resistance, particularly in sedentary individuals and those undergoing lifestyle interventions [14,15,16]. These findings support the hypothesis that TDH dysregulation is a shared oxidative mechanism underlying both sarcopenia and obesity.

In parallel, ischemia-modified albumin (IMA), a serum biomarker formed under conditions of oxidative stress and hypoxia, has also gained attention as a relevant indicator of oxidative damage [17]. IMA levels are elevated in numerous oxidative stress-related conditions and have been shown to rise in sarcopenia and osteosarcopenia, correlating with alterations in TDH [13].

Despite these insights, the role of TDH and IMA in SO remains poorly understood. Given the pathophysiological relevance of redox imbalance in both obesity and sarcopenia, evaluating TDH and IMA together may provide a more comprehensive understanding of oxidative stress in SO and aid in identifying individuals at elevated risk.

Therefore, the present study aims to investigate the association between SO and oxidative stress parameters, specifically thiol/disulfide homeostasis and ischemia-modified albumin levels, in older adults. Elucidating this relationship may enhance our understanding of redox-driven mechanisms in SO and potentially support the development of antioxidant-focused prevention or treatment strategies in geriatric care.

## 2. Materials and Methods

### 2.1. Study Design and Population

This cross-sectional study was conducted among adults aged 65 years and older who presented consecutively to the geriatric outpatient clinic. Based on an expected SO prevalence of 15% in older obese adults and a margin of error of 7%, a minimum of 100 participants was required to achieve a 95% confidence level [3]. A total of 200 patients were screened, and 132 obese individuals were enrolled. Participants were excluded if they had active inflammatory or infectious diseases; malignancy; severe renal or hepatic dysfunction; neurodegenerative or musculoskeletal conditions affecting mobility; recent surgery or hospitalization; use of corticosteroids, immunosuppressants, or antioxidant supplements; or an inability to perform physical performance tests.

Obesity screening and SO diagnosis were conducted in accordance with the consensus definition proposed by the European Society for Clinical Nutrition and Metabolism (ESPEN) and the European Association for the Study of Obesity (EASO) [18]. For screening, obesity was identified based on a Body Mass Index (BMI) ≥30 kg/m^2^ or waist circumference ≥90 cm in males and ≥80 cm in females [19,20]. The diagnosis of SO required evidence of both impaired muscle function, decreased muscle mass, and excess adiposity. Excess adiposity was defined as body fat percentage >31% in males and >43% in females [21], while impaired muscle function and mass were indicated by handgrip strength <27 kg in males and <16 kg in females [22], chair stand test duration ≥17 s [23], and skeletal muscle mass to weight ratio (SMM/W) ≤0.370 in males and ≤0.276 in females [24].

The study protocol was reviewed and approved by the local ethics committee with the reference number GO:21/851. All procedures involving human participants were conducted in accordance with the ethical standards of the institutional and national research committee, and with the 1964 Helsinki Declaration and its later amendments. Written informed consent was obtained from all participants prior to enrollment.

### 2.2. Clinical and Functional Assessments

All participants underwent a comprehensive geriatric assessment that included the Katz Index of Independence in Activities of Daily Living (ADL) [25,26], Lawton–Brody Instrumental Activities of Daily Living (IADL) [27,28], Clinical Frailty Scale (CFS) [29], Mini Nutritional Assessment Short Form (MNA-SF) [30,31], Yesevage Geriatric Depression Scale (GDS) [32,33], and Standardized Mini-Mental State Examination (SMMSE) [34]. Additionally, comorbidities of the patients were recorded, and the Charlson Comorbidity Index (CCI) was calculated [35].

### 2.3. Anthropometric and Body Composition Measurements

Height was measured using a stadiometer with participants standing barefoot and erect, with the head positioned in the Frankfurt plane. Weight was recorded using a calibrated digital scale with participants in light clothing and no shoes. Body Mass Index (BMI) was calculated as weight in kilograms divided by the square of height in meters (kg/m^2^). Waist circumference was measured midway between the lowest rib and the iliac crest using a non-elastic measuring tape, while the participant was standing and breathing out gently, following the standardized anthropometric protocols [36].

Fat mass percentage was assessed using a multifrequency bioelectrical impedance analysis (BIA) device (Bodystat QuadScan 4000, Douglas, Isle of Man, UK), with standardized pre-measurement conditions including fasting and avoidance of strenuous activity or caffeine for 12 h. Skeletal muscle mass (SMM) was derived from BIA readings with the equation of fat-free mass × 0.566 and further normalized by body weight to calculate the skeletal muscle mass index (SMM/W) [37].

### 2.4. Muscle Strength

Handgrip strength was measured using a calibrated handheld dynamometer (Takei TKK 5401 Grip-D Dynamometer, Takei Scientific Instruments, Niigata, Japan). Participants were seated with the elbow flexed at 90°, the wrist maintained in a neutral position, and the forearm resting on the armrest of the chair and instructed to squeeze maximally. Three measurements were taken from the dominant hand, and the maximum value was recorded.

The chair stand test (five-times sit-to-stand) was used to assess lower extremity function. Participants were asked to rise from a standard chair and sit down five times as quickly as possible without using their arms. The total time taken was recorded in seconds.

### 2.5. Measurement of Oxidative Stress Parameters

Venous blood samples were collected from all participants in the morning after an overnight fast to minimize metabolic variability. Samples were drawn under standardized conditions using sterile vacutainer tubes and centrifuged at 1600× *g* for 10 min, and the resulting serum aliquots were stored at –80 °C until analysis to preserve biochemical integrity. Serum levels of native thiol and total thiol were measured using the fully automated colorimetric spectrophotometric method originally developed and validated by Erel and Neşelioğlu [38]. This method relies on the reducibility of dynamic disulfide bonds into functional thiol groups under controlled conditions, followed by quantification with DTNB (5,5′-dithiobis-(2-nitrobenzoic acid)). Disulfide levels were calculated indirectly using the following formula: Disulfide = (Total Thiol − Native Thiol)/2. The disulfide/native thiol ratio and disulfide/total thiol ratio were derived to assess the oxidative status and redox equilibrium. Elevated disulfide ratios indicate a shift toward oxidized states, reflecting increased oxidative stress and impaired antioxidant capacity [38].

IMA levels were assessed using the albumin cobalt binding test, as described by Bar-Or et al. [39]. In this assay, serum samples are incubated with cobalt chloride, which binds to the N-terminus of unmodified albumin. In the presence of oxidative stress or ischemia, structural changes in albumin reduce its cobalt-binding capacity. Dithiothreitol (DTT) is added to quench excess cobalt ions, and the final absorbance is measured at 470 nm using a spectrophotometer. The results are expressed in absorbance units.

### 2.6. Statistical Analyses

Data were analyzed using SPSS version 27 (IBM Corp., Armonk, NY, USA). Continuous variables were presented as mean ± standard deviation or median with interquartile range, and categorical variables as frequencies and percentages. Group comparisons were performed using the independent samples t-test or Mann–Whitney U test for continuous variables, and chi-square test for categorical variables. To identify independent predictors of SO, a binary logistic regression analysis was performed using the backward stepwise likelihood ratio method. Variables included in the initial model were age, sex, Katz ADL, Lawton–Brody IADL, SMMSE, CFS, CCI, and oxidative stress parameters (native thiol, total thiol, disulfide/native thiol ratio, and disulfide/total thiol ratio). Variables that did not contribute significantly to the model were eliminated in successive steps, and the final model included only those with a statistically significant association with the outcome. Model fit was assessed using the Hosmer–Lemeshow goodness-of-fit test, and predictive accuracy was evaluated via classification tables and Nagelkerke R^2^ values.

Additionally, a receiver operating characteristic (ROC) curve analysis was conducted to assess the diagnostic performance of the disulfide/native thiol ratio in predicting SO. The area under the curve (AUC) was calculated with 95% confidence intervals (CI). The optimal cut-off point was determined using the Youden index, and corresponding sensitivity and specificity values were reported. Receiver operating characteristic (ROC) curve analysis was performed using MedCalc Statistical Software (MedCalc Statistical Software version 19.2.6, Ostend, Belgium). A *p*-value < 0.05 was considered statistically significant for all analyses.

## 3. Results

A total of 200 consecutive patients aged 65 years and older were screened for the study, and 132 obese individuals met the inclusion criteria and were enrolled. The median age of the overall study population was 73 years (69–78), and 64.4% (*n* = 85) of the participants were female. Among them, 20 patients (15.2%) were diagnosed with SO based on the ESPEN/EASO diagnostic consensus.

### 3.1. Patient Characteristics

The median age of participants with SO was significantly higher than those without SO [78 (71–87) vs. 73 (68–76) years, *p* = 0.007]. Although females were more frequent in both groups, the difference in sex distribution was not statistically significant (*p* = 0.14). Body weight and BMI were significantly higher in the SO group compared to the non-SO group (weight: 85.35 ± 11.82 kg vs. 77.93 ± 12.94 kg, *p* = 0.018; BMI: 33.2 vs. 29.4 kg/m^2^, *p* = 0.026). Waist circumference was also significantly greater among females with SO (*p* < 0.001), while no significant difference was observed in males (*p* = 0.59).

In terms of muscle strength and function, patients with SO had significantly lower handgrip strength in both sexes (female: *p* = 0.001; male: *p* = 0.007) and a longer chair stand test duration (*p* = 0.029). Although SMM did not differ significantly between groups, the SMM/W was markedly lower in both females and males with SO (*p* < 0.001 for both). Fat mass percentages were significantly higher in the SO group across both sexes (*p* < 0.001) (Table 1).

### 3.2. Comprehensive Geriatric Assessment

Lawton–Brody IADL scores were significantly reduced in the SO group (*p* = 0.002), and patients were frailer, as indicated by higher CFS scores (*p* = 0.007). Katz ADL and SMMSE scores showed trends toward lower function in the SO group but did not reach statistical significance. Nutritional status and depressive symptoms were similar between groups based on MNA-SF and GDS, respectively (Table 1).

### 3.3. Oxidative Stress Parameters

Native thiol levels were significantly lower in the SO group (263.55 ± 74.51 μmol/L) compared to the non-SO group (307.55 ± 57.77 μmol/L, *p* = 0.003). Total thiol levels were also reduced in SO patients (283.71 ± 69.59 μmol/L vs. 339.21 ± 60.59 μmol/L, *p* < 0.001). Although disulfide levels did not differ significantly between groups (*p* = 0.74), oxidative stress indices were elevated. Disulfide/native thiol was higher in the SO group [median 5.62% (5.09–7.99)] vs. non-SO [5.15% (4.61–5.88)], *p* = 0.009. No statistically significant difference was found in IMA levels between groups (0.88 vs. 0.84 mg/dL, *p* = 0.13) (Table 1).

### 3.4. Predictors of Sarcopenic Obesity

To determine the independent predictors of SO, a binary logistic regression analysis was performed using a backward stepwise likelihood ratio method. The initial model included variables such as age, sex, Katz ADL, Lawton–Brody IADL, SMMSE, CFS, CCI, and oxidative stress parameters (native thiol, total thiol, disulfide/native thiol ratio, and disulfide/total thiol ratio). After backward stepwise logistic regression, the final model (Model 8) retained age, native thiol, total thiol, and disulfide/native thiol ratio as statistically significant predictors of SO, demonstrating good fit according to the Hosmer–Lemeshow test. (Table 2).

Age was positively associated with SO (OR = 1.094, 95% CI: 1.006–1.189, and *p* = 0.035), and the disulfide/native thiol ratio was significantly associated with increased odds of SO (OR = 5.713, 95% CI: 1.076–30.329, and *p* = 0.041).

### 3.5. Predictive Accuracy of Disulfide/Native Thiol Ratio for Sarcopenic Obesity

**Figure 1:**
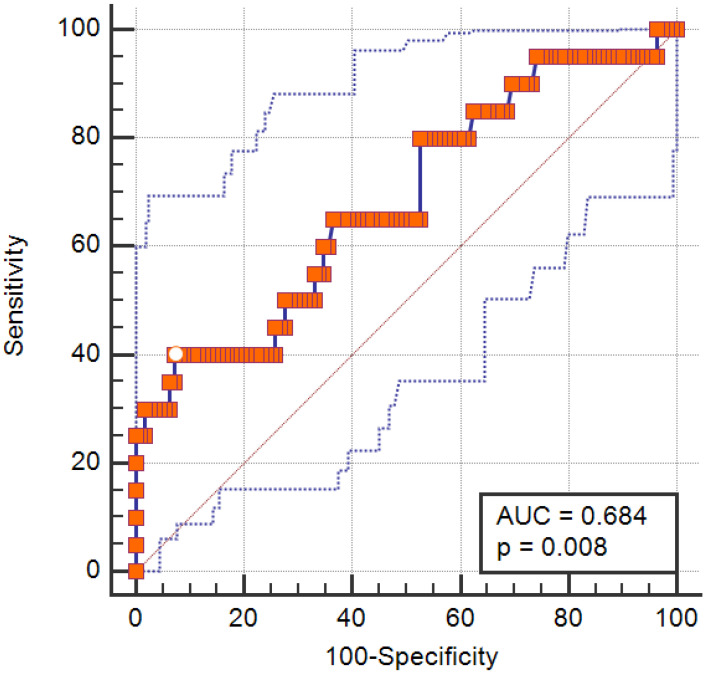
Receiver operating characteristic curve demonstrating predictive performance of the disulfide/native thiol ratio in predicting SO. The solid blue line represents the ROC curve, the red diagonal line indicates the reference line, and the red square markers represent the coordinates of the curve. The white dot indicates the optimal cutoff point based on the Youden Index.

The diagnostic performance of the disulfide/native thiol ratio in predicting SO was evaluated using an ROC curve. The AUC was 0.684 (95% CI: 0.597–0.762; *p* = 0.008), indicating moderate discriminatory ability (Figure 1). The optimal cut-off value identified by the Youden index was >6.63, which yielded a sensitivity of 40.0% and a specificity of 92.86%.

## 4. Discussion

In this study, we investigated the association between TDH, IMA, and SO in a cohort of obese older adults. We found that individuals with SO exhibited significantly lower native and total thiol levels and higher disulfide/native thiol and disulfide/total thiol ratios compared to their non-SO counterparts, indicating a state of elevated oxidative stress. Furthermore, the disulfide/native thiol ratio was identified as an independent predictor of SO in logistic regression analysis, and ROC curve analysis demonstrated that this ratio had moderate discriminatory power for predicting SO (AUC = 0.684, *p* = 0.008).

Our findings align with prior literature suggesting that oxidative stress plays a critical role in the pathogenesis of both sarcopenia and obesity [12,13,14,15]. Erel and Neşelioğlu’s method for evaluating dynamic TDH provides a sensitive marker for assessing redox status in various clinical settings, and alterations in TDH have been associated with metabolic diseases, frailty, urinary incontinence, and sarcopenia [10,12,40,41]. In particular, Özsürekci et al. reported significantly decreased thiol levels and increased disulfide ratios in older adults with sarcopenia, supporting our observation that a shift toward an oxidized state is characteristic of muscle degradation and dysfunction in aging populations [12].

Moreover, to the best of our knowledge, this is the first study to specifically investigate TDH in the context of SO—a condition that combines the metabolic complications of obesity with the functional and structural muscle loss characteristic of sarcopenia. Emerging evidence supports the hypothesis that oxidative stress plays a central mechanistic role in the development of SO, acting as a common pathway linking adipose tissue expansion, chronic inflammation, and muscle catabolism [7,8,9]. Gonzalez et al. highlighted that elevated reactive oxygen species (ROS) levels contribute to mitochondrial dysfunction, insulin resistance, and inflammation, all of which accelerate muscle protein degradation while promoting fat accumulation [8].

Similarly, Jung reviewed how oxidative stress not only impairs muscle regeneration and differentiation but also enhances adipogenic pathways, thereby favoring the simultaneous progression of sarcopenia and obesity. These redox alterations reduce skeletal muscle quality and quantity while exacerbating metabolic derangements—creating a vicious cycle that perpetuates SO [9]. Furthermore, Li et al. emphasized that the imbalance between fat mass and lean mass contributes to local and systemic oxidative damage, further aggravating muscle loss and functional decline [7].

While disulfide levels alone did not differ significantly between the SO and non-SO groups in our study, the elevated disulfide/native thiol ratio observed in SO patients suggests that oxidative shifts in dynamic thiol/disulfide balance, rather than absolute thiol or disulfide concentrations, may better reflect the underlying redox imbalance. Together, these results support the conceptual framework that oxidative stress is not merely a byproduct but a driving factor in the pathogenesis of SO, reinforcing the clinical importance of monitoring redox biomarkers in this vulnerable population.

Interestingly, although IMA levels were slightly higher in patients with SO, the difference did not reach statistical significance in our study. This observation is consistent with the findings of Özsürekçi et al. and İleri et al., both of whom also reported no significant differences in IMA levels between individuals with sarcopenia or osteosarcopenia [12,13]. These results suggest that, despite the role of oxidative stress in the pathophysiology of SO, IMA may not be sufficiently sensitive or specific to detect redox imbalance in this context.

IMA is known to increase in response to acute ischemic events and oxidative tissue injury [42]. However, its diagnostic performance appears to be more reliable in acute and overt ischemic states rather than in chronic, low-grade inflammatory conditions like SO [17]. Taken together, these insights indicate that while IMA reflects oxidative alterations, its clinical utility in SO is likely limited, especially when compared to more dynamic and sensitive redox biomarkers such as TDH parameters.

Sex-based physiological differences may also influence the oxidative stress response observed in SO [7,8]. In this study, although the overall sample was predominantly female, subgroup analysis was limited by the small number of male subjects with SO. Females generally have higher fat mass and lower skeletal muscle mass compared to males, even after adjusting for age and BMI, which could contribute to differential redox profiles [5,43]. Estrogen is known to exert antioxidant effects, whereas postmenopausal hormonal decline may exacerbate oxidative imbalance in older women [44,45]. Moreover, sex-specific differences in inflammatory pathways, mitochondrial function, and adipokine signaling may modulate TDH [46]. Future studies with balanced sex distributions are needed to explore whether the predictive value of TDH parameters varies by sex and to clarify potential sex-specific oxidative mechanisms in the pathogenesis of SO.

Several limitations of this study should be noted. First, the cross-sectional design limits our ability to infer causality between oxidative stress parameters and SO. Second, although we used BIA for muscle and fat mass assessment, more precise methods such as dual energy X-ray absorptiometry were not available. Third, the relatively small number of patients with SO (*n* = 20) may limit statistical power, particularly for subgroup analyses. Lastly, despite controlling for relevant confounders in multivariate analyses, residual confounding due to unmeasured variables (e.g., dietary antioxidant intake, inflammatory markers) cannot be excluded.

## 5. Conclusions

The findings of this study have important clinical implications. TDH, particularly the disulfide/native thiol ratio may serve as a practical, cost-effective biomarker for identifying older adults at risk for SO. Integrating oxidative stress profiling into routine geriatric assessment could improve early detection and prompt targeted interventions, such as antioxidant therapy, resistance training, and nutritional optimization.

Future studies should adopt longitudinal designs to determine whether changes in TDH precede the development of SO and whether interventions that restore redox balance can mitigate its onset or progression. Moreover, combining TDH with other biomarkers (e.g., inflammatory cytokines, mitochondrial markers) may enhance diagnostic accuracy and yield mechanistic insights.

In conclusion, this study demonstrates that older adults with SO exhibit disrupted TDH, marked by decreased thiol levels and increased oxidative stress ratios. The disulfide/native thiol ratio emerged as an independent predictor of SO and showed moderate accuracy in ROC analysis, supporting its potential utility as a biochemical indicator of SO. These results contribute to the growing evidence that oxidative stress is a key mechanism underlying SO and warrant further investigation into redox-based diagnostic and therapeutic strategies in geriatric populations.

## Figures and Tables

**Table 1:** Demographic features, clinical characteristics, comprehensive geriatric assessment scores, and oxidative stress parameters stratified by sarcopenic obesity status.

	SO (*n* = 20)	Non-SO (*n* = 112)	*p*
Age, years, IQR	78 (71–87)	73 (68–76)	**0.007**
Sex, female, *n*, %	10 (50.0%)	75 (67.0%)	0.14
Height, cm, ±SD	160.30 ± 10.45	160.12 ± 8.82	0.93
Weight, kg, ±SD	85.35 ± 11.82	77.93 ± 12.94	**0.018**
BMI, kg/m^2^, IQR	33.2 (28.35–38.77)	29.40 (27.02–32.87)	**0.026**
Waist circumference, cm, ±SD			
Female	112.60 ± 11.04	99.97 ± 8.78	**<0.001**
Male	103.90 ± 9.80	102.16 ± 8.99	0.59
Handgrip strength, kg, IQR			
Female	13.9 (10.6–15.4)	20.0 (15.6–23.1)	**0.001**
Male	21.1 (18.7–25.8)	(29.9 (25.3–34.6)	**0.007**
Chair stand, s, IQR	17.26 (12.52–18.44)	14.25 (11.98–16.99)	**0.029**
SMM, kg, IQR			
Female	22.85 (18.91–24.72)	23.59 (21.29–25.97)	0.29
Male	30.19 (26.31–34.74)	31.89 (29.94–36.40)	0.094
SMM/W, IQR			
Female	0.262 (0.258–0.269)	0.308 (0.290–0.332)	**<0.001**
Male	0.360 (0.337–0.370)	0.400 (0.382–0.418)	**<0.001**
Fat mass, %, IQR			
Female	53.55 (52.32–54.50)	45.60 (41.40–49.00)	**<0.001**
Male	36.70 (34.52–40.50)	28.90 (26.15–32.25)	**<0.001**
Comprehensive Geriatric Assessment			
Katz ADL, IQR	5 (4–6)	6 (5–6)	**0.059**
Lawton–Brody IADL, IQR	6 (4–8)	8 (8–8)	**0.002**
CFS, IQR	5 (3–5)	3 (3–4)	**0.007**
GDS, IQR	3 (0–6)	3 (0–6)	0.90
SMMSE, IQR	24 (21–28)	27 (24–29)	0.092
MNA-SF, IQR	13 (11–14)	14 (12–14)	0.56
Charlson comorbidity index, IQR	5 (3–6)	4 (3–5)	0.053
Oxidative Stress Parameters			
Native thiol, μmol/L ±SD	263.55 ± 74.51	307.55 ± 57.77	**0.003**
Total thiol, μmol/L ±SD	283.71 ± 69.59	339.21 ± 60.59	**<0.001**
Disulfide, μmol/L, IQR	15.05 (14.3–17.55)	15.67 (14.26–17.0)	0.74
Disulfide/Nativethiol, IQR	5.62 (5.09–7.99)	5.15 (4.61–5.88)	**0.009**
Disulfide/Totalthiol, IQR	5.05 (4.62–6.88)	4.67 (4.22–5.26)	**0.009**
IMA, mg/dL, IQR	0.88 (0.72–0.98)	0.84 (0.68–0.92)	0.13

ADL: Katz Index of Independence in Activities of Daily Living, BMI: Body Mass Index, CFS: Clinical Frailty Scale, GDS: Yesevage Geriatric Depression Scale, IADL: Lawton–Brody Instrumental Activities of Daily Living, IMA: ischemia-modified albumin, IQR: interquartile range, MNA-SF: Mini Nutritional Assessment Short Form, SD: standard deviation, SMMSE: Standardized Mini Mental State Examination, SMM: skeletal muscle mass, SMM/W: skeletal muscle mass index, and SO: sarcopenic obesity. Bolded values indicate statistically significant results (*p* < 0.05).

**Table 2:** Logistic regression analysis of the independent factors associated with sarcopenic obesity (only the final step is shown in the table due to utilizing the backward stepwise likelihood ratio method).

		SO		
		Odds Ratio	95% CI	*p*-Value
Model 8	Age	1.09	1.006–1.189	**0.035**
	Native thiol	1.35	0.94–1.93	0.095
	Total thiol	0.75	0.54–1.04	0.093
	Disulfide/Nativethiol	5.71	1.07–30.32	**0.041**

Hosmer–Lemeshow goodness-of-fit test *p* = 0.45. CI: confidence interval, SO: sarcopenic obesity. Model 8 refers to the final multivariate logistic regression model obtained through stepwise backward elimination. Bolded values indicate statistically significant results (*p* < 0.05). The Hosmer–Lemeshow test was used to assess model calibration (*p* > 0.05 indicates good fit).

## Data Availability

The data that support the findings of this study are available from the corresponding author upon reasonable request.
